# Young adults from disadvantaged groups experience more stress and deterioration in mental health associated with polycrisis

**DOI:** 10.1038/s41598-024-59325-8

**Published:** 2024-04-16

**Authors:** Weronika Kałwak, Dorota Weziak-Bialowolska, Anna Wendołowska, Karolina Bonarska, Katarzyna Sitnik-Warchulska, Anna Bańbura, Dorota Czyżowska, Aleksandra Gruszka, Małgorzata Opoczyńska-Morasiewicz, Bernadetta Izydorczyk

**Affiliations:** 1 Institute of Psychology, Faculty of Philosophy, Jagiellonian University, ul. Romana Ingardena 6, 30-060 Kraków, Poland; 2 Department of Quantitative Methods and Information Technology, Kozminski University, ul. Jagiellonska 57/59, 03-301 Warsaw, Poland; 3 Human Flourishing Program, Institute for Quantitative Social Science, Harvard University, 12 Arrow St, Cambridge, MA 02138 USA; 4 Institute of Applied Psychology, Faculty of Management and Social Communication, Jagiellonian University, ul. Prof. Stefana Łojasiewicza 4, 30-348 Kraków, Poland

**Keywords:** Polycrisis, College students, Young adults, Disadvantaged populations, Mental health, Cumulative stress, Psychology, Environmental social sciences

## Abstract

The recent polycrisis (COVID-19, Ukraine war, climate change, economic crisis) has been associated with mental health through cumulative stress, with young people being particularly vulnerable. We surveyed 403 college students from Poland to examine their psychological responses to the experienced crises. The results showed that polycrisis was associated with worse mental health of college students from disadvantaged groups (based on gender, sexual orientation, and financial situation) compared to other college students, in four areas: sense of proximity to the crises, stress caused by the crises, sense of responsibility for mitigating the crises, and experiencing everyday moral dilemmas regarding the crises. These young adults also suffered more in terms of negative affectivity, depressive symptoms, and subjective physical and mental health. Our findings suggest that when discussing public mental health perspectives, it is important to consider consequences of cumulative stress and its greater impact on young people from disadvantaged groups.

## Introduction

This paper presents exploratory findings concerning the susceptibility of young adults to the impact of stress and increased risk of experiencing poorer mental health when confronted by the ongoing polycrisis. The concept of polycrisis refers to the multiple global and local crises (i.e. COVID-19 pandemic and its health consequences; global climate emergency; ongoing wars, especially the war in Ukraine; and the widespread cost-of-living crisis) co-occurring and inter-related in a way that exacerbates their degrading effects and causes convergent harm^1–8^. In recent years, the concept of polycrisis—proposed by a historian, Adam Tooze, and a theorist of complexity, Edgar Mori—has been gaining popularity within and outside the academic community. However, it was initially accompanied by some doubts resulting from its weak scientific basis. Although complex, systemic, and convergent phenomena pose challenges for investigation, a growing body of scientific evidence has conceptualized polycrisis in causal terms and built research frameworks on the overlapping consequences of multiple crises^1–8^. The concept of polycrisis (including a consideration of multiple crises as inseparable threats that must be addressed together) is increasingly used by respected international organizations, including the World Health Organization and United Nations Children’s Fund (UNICEF), which recognize polycrisis as a global threat to human health^9–11^. The widespread impact of current global and local crises on mental health is one of the most important challenges to public health; this topic is usually discussed in the context of various vulnerabilities^7^^,^^12–15^. While young age is generally considered a factor of vulnerability, this study addressed the situation of young people in times of polycrisis that creates conditions of multiplied vulnerability^12^^,^^13^^,^^15^^,^^16^.

While the worsening mental health of young people has been considered a global challenge to public health, region-specific knowledge is the most effective for designing tailored interventions^17^. A recent communication from the European Commission on a comprehensive approach to mental health warns that Europe has been witnessing an unprecedented mental health deterioration among young people^16^. The responsibility for global crises in recent years has been ascribed particularly to the COVID-19 pandemic; displacement and trauma resulting from the war in Ukraine; alarming vision of climate change; and poverty, inequalities, and social exclusion^4^^,^^6^^,^^12^^,^^16^. Despite the relatively high resources invested in mental healthcare and the availability of mental health services in Europe, mental health conditions remain a significant burden for young people in this region, where suicide has become the second most prevalent cause of death in this age group^16^^,^^17^.

Since 2020, numerous empirical studies, reviews, and meta-analyses have shown the prevalent negative impact of COVID-19 on mental health worldwide. For example, 36% of the general population reported poor mental health^18^. A recent systematic review of longitudinal studies conducted in 11 countries showed an increase in distress and negative affect, and a decrease in mental health among young people, especially teenagers and young adults^19^. In Europe, for example, during the pandemic, depression incidents in young people more than doubled^16^, eating disorders increased considerably, especially in young females, and feelings of loneliness were reported by 20% of young people^16^^,^^17^. A longitudinal study of German children and teenagers revealed a significant decrease in health-related quality of life and an almost doubled increase in overall mental health problems. Most importantly, although the numbers decreased three years after the pandemic, they did not return to pre-pandemic levels, and young people reported concerns related to other current crises^20^.

Since before the pandemic, mental health has been extensively discussed in the context of climate change and environmental degradation, with the younger generation being perceived as more affected by these occurrences and facing a greater risk of subsequent mental health issues^16^^,^^21^. While most of the empirical studies thus far have been conducted outside Europe and usually in the regions already deeply affected by climate change-related extreme weather events and natural disasters (e.g. children experiencing post-traumatic stress disorder; PTSD following Australian wildfires), preliminary analyses of the direct and indirect impacts of environmental degradation on young people’s mental health have been conducted in the European region^22^. Importantly, climate change awareness, environmental concerns, anticipatory ecological emotions (e.g. climate change anxiety), engagement in pro-environmental individual and collective actions, and exposure to environmental communication are important value-laden psychological factors that mediate the impact of environmental degradation on young people’s mental health in the Global North, including Europe^12^^,^^23^^,^^24^. This is the case even if personal experiences of environmental degradation, natural disasters, and extreme weather events are lacking for most young people^21^^,^^25^^,^^26^. Young people’s sense of responsibility for mitigating the climate crisis in the face of hopelessness, helplessness, and witnessing the inaction of decision-makers, as well as experiencing moral dilemmas related to climate change when making everyday decisions and plans, may increase the burden of climate change-related distress due to moral stress^27^^,^^28^.

The mechanisms by which climate change impacts mental health are complex and multidimensional. While research on mental health in terms of causality is generally challenging, it is even more challenging when environmental stressors such as crises are taken into consideration. Not only environmental awareness or heatwaves but also socio-political tensions or economic burdens resulting from global climate change may affect human health and psychological conditions^12^. Furthermore, various current global and regional crises are constantly interacting with the climate crisis, and the burdens they may cause not only interact but also cumulate^2–5^^,^^12^. Consequently, the term polycrisis has been coined and used in this context to emphasize the interconnectedness of these crises^4^^,^^6^. Therefore, they should not be considered in isolation when their consequences on mental health and stress are discussed^12^^,^^29^. The interconnected crises may be hypothesized to cause cumulative stress and burden. The well-established notion of cumulative stress incorporates a significant role of environmental stressors (including those related to crises) alongside individual stressors and predispositions^30–34^. The current polycrisis may serve as a case study that helps understand future pandemics, social and economic troubles, civil and armed conflicts, and the mass migration expected to occur in the future due to the deepening climate crisis^4^.

It is well recognized that the adverse consequences of crises and destabilization of any kind in global and local social and natural environments preliminarily affect regions, communities, groups, and individuals that are the most vulnerable and underprivileged (e.g. the lower-income countries in the Global South or ethnic minority communities)^12^^,^^13^^,^^35^. In the case of individuals, vulnerability to mental health deterioration may be due to various disadvantages, often defined in terms of sex and gender, socioeconomic status, or pre-existing health conditions^12^^,^^13^^,^^35–37^. Thus, the female gender, non-heterosexual orientation, non-binary sexual identity, lower socioeconomic status, pre-existing psychiatric diagnosis, disability, migration, and minority backgrounds have been associated with generally worse mental health among individuals (i.e. due to marginalization, social exclusion, discrimination, and violence)^15^^,^^38–52^^,^^78^. These factors often co-occur and create conditions of intersectionality (e.g. females have a lower economic status or transgender individuals suffer from more mental health conditions; Refs.^13^^,^^15^^,^^53^). Especially within disadvantaged groups, young age is considered an additional vulnerability factor^12^^,^^15^^,^^16^^,^^54^; this may result from the immature coping mechanisms of a still-developing individual who, in the face of a crisis, meets challenges that are difficult to cope with, even as an adult. Second, young people usually have less access to resources that help protect individuals’ mental health and maintain resilience^55^. Therefore, young age, when co-existing with other vulnerability factors, creates a multiplied risk for mental health issues in times of peace and stability, let alone in an era of polycrisis, as shown separately through the COVID-19 pandemic, climate crisis, economic crisis, and civil and armed conflicts^12^^,^^16^^,^^21^^,^^29^^,^^54^. Third, besides the general deterioration in living conditions during unstable times, a brutalization of social relationships and habits is observed in the context of any crisis; consequently, discrimination and violence have escalated^56–58^. In these circumstances, vulnerable and disadvantaged young people may find it even more difficult to live in their social and natural environment while maintaining good mental health. This may also add to the cumulative stress of the polycrisis.

Existing literature contains information about the worsening mental health conditions of young people (including young adults), and young age is considered a vulnerability factor. Previous studies have provided evidence for a greater risk of deteriorating mental health and higher stress levels in individuals belonging to disadvantaged groups, and this relationship is emphasized in young people. The novelty of the present study lies in addressing stress levels and the mental health of young adults (being college students) in times of cumulative stressors arising in the context of polycrisis.

### Aims of the study

While polycrisis is expected to affect the mental health of young adults, we examined how the psychological responses to the four kinds of crises are associated with their mental health. In particular, we examined whether these responses are different among college students from disadvantaged groups (i.e. females and other genders, individuals with non-heterosexual orientation, and individuals in unfavorable financial situations) compared to other college students. We focused on four recently co-occurring crises—the COVID-19 pandemic, climate emergency, war in Ukraine, and the recent cost-of-living crisis—which we examined via four areas of psychological responses to the crises: sense of proximity to the crises, stress caused by them, sense of responsibility for mitigating them, and everyday moral dilemmas regarding them.

## Methods

### Data

Using convenience sampling, 403 college students were recruited for this study. The minimum sample size was calculated based on the number of students in Poland, which was approximately 1,200,000. The inclusion criteria were: aged between 21 and 24 years (year of birth: 1999–2002), Polish residence (including living in Poland from 2019 to 2023), and being a university student. The exclusion criteria were: not having a Polish residence from 2019 to 2023, aged below 19 and above 24 years, and not being a university student. The age criteria were set to investigate emerging adults who graduated from high school and entered university education in 2020 (the most severe stage of the COVID-19 crisis).

Data were collected between February and May 2023. An online survey designed in Qualtrics was used. An anonymous link to the survey was distributed among students at various universities and faculties in Poland. Respondents were informed of the survey’s aim, instructions for participation, and the option to opt out at any time. Participation in this study was voluntary. All methods were performed per the relevant guidelines and regulations.

In the analytical sample, the proportion of males, females, and other genders was 15.9%, 80.8%, and 3.2%, respectively. First-, second-, third-, fourth-, and fifth-grade students accounted for 18.9%, 28.8%, 31.3%, 13.2%, and 7.9% of the participants, respectively. The participants were mostly heterosexual (70.5%), living either with parents (28.3%) or roommates (29.5%), at least partially financially independent (54.6%), and working either occasionally or regularly (30.0% and 42.0%, respectively).

### Measures

#### Psychological response to polycrisis

The psychological responses to each of the four crises were assessed with respect to four areas:

- Sense of proximity to each crisis (responses: 1 = I have never heard about it; 2 = I know it only from media reports; 3 = Someone I know was affected by it; 4 = I was affected by it),
- Stress caused by each crisis (1 = I do not feel stressed about it at all; 2 = I rather do not feel stressed about it; 3 = I feel stressed about it a little bit; 4 = I feel stressed about it very much),
- Responsibility felt for mitigating each crisis (1 = I do not feel responsible for it at all; 2 = I rather do not feel responsible for it; 3 = I feel somewhat responsible; 4 = I feel highly responsible),
- Experiencing everyday moral dilemmas regarding each crisis (1 = never, 2 = rarely, 3 = often, 4 = daily).

This resulted in 16 crisis-related variables (four crises × four areas). For each area, a summary index of effects was constructed (by summing up responses across the four crises) to assess the cumulative response to the crises in each area. Consequently, four variables were constructed: sense of proximity to crises, stress due to crises, sense of responsibility for mitigating the crises, and everyday moral dilemmas related to crises (range 4–16; the higher the score, the more affected the individuals).

#### Self-assessment of physical and mental health

A two-item health subscale based on the Flourishing Index was used (Cronbach’s alpha = 0.84^59^). This instrument has been conceptualized to capture complete well-being and comprises five two-item scales: happiness and life satisfaction, meaning and purpose in life, character and virtue, social connectedness, and health. The psychometric properties of the instrument have been positively evaluated in culturally distinct populations^60^ and its single items have proved useful in various studies on well-being and health (e.g. Refs.^61–63^. Respondents respond to two questions: In general, how would you rate your physical health? 0 = Poor, 10 = Excellent; How would you rate your overall mental health? 0 = Poor, 10 = Excellent. The total score is derived as a simple average. Higher scores reflect better self-assessment of health.

#### Negative affect

The negative affectivity subscale of the Polish adaptation of the Personality Inventory for the International Classification of Diseases 11th Revision (ICD-11; PiCD) was used^64^. This subscale comprises 12 items. Respondents respond on a five-point Likert scale (1 = strongly disagree, 5 = strongly agree). The total score for negative affectivity is computed as the simple average of the 12 items (Cronbach’s alpha = 0.87). Higher scores reflect stronger negative affectivity.

#### Depression

Depression was assessed using the Polish version of the Brief Symptoms Inventory 18 (BSI-18^65^). The BSI-18 comprises 18 items, including six for depression assessment, each rated on a five-point scale (0 = Not at all, 4 = Extremely). Respondents provide information on the frequency of some of the core symptoms of depression experienced over the preceding seven days. The total depression score is derived as a simple average of the depression items (Cronbach’s alpha = 0.85). Higher scores correspond to more severe symptoms.

#### Disadvantaged groups

These groups were defined according to gender (male vs. female vs. other), sexual orientation (heterosexual vs. non-heterosexual—including homosexual, bisexual, asexual, and other), and financial situation (self-assessment of financial situation in the last year dichotomized into favorable—very good, good, fair—and difficult—very difficult, difficult, moderately difficult).

### Statistical analysis

To investigate the psychological response to polycrisis, we examined the co-occurrence of responses to the crises under study, specifically in terms of sense of proximity, induced stress, perceived responsibility, and experienced moral dilemmas. This was achieved by reporting the prevalence of experiences related to a certain number of crises within each area.

To evaluate the associations between polycrisis responses and demographic characteristics (used to define disadvantaged groups), along with potential mental health confounding factors, we employed an outcome-wide analysis as suggested by VanderWeele et al.^66^. We scrutinized 20 outcomes in total, which consisted of 16 individual crisis-related variables and four cumulative crisis variables. This all-encompassing analysis allowed us to discern the structure of associations that might otherwise remain hidden if only a single outcome was analyzed. Moreover, this strategy promotes the publication of non-significant and negative results. These results, often absent in published research, help to counteract the so-called publication bias, as noted by Fanelli^67^. For 16 individual crisis-related outcomes, the ordered logit regression model was applied and odds ratios were reported. For four cumulative crisis outcomes, the linear regression model was applied and unstandardized regression coefficients were reported.

### Ethics approval

This study was approved by the Research Ethics Committee of the Faculty of Philosophy of Jagiellonian University (no. 221.0042 26-23) and registered with Clinical Trials (no. NCT05930652).

### Consent

Informed consent was obtained from all the participants of this study.

## Results

Table 1 presents the descriptive statistics for the individual crisis-related variables (categorical variables). Table 2 presents the descriptive statistics for the cumulative psychological responses to the crises and for mental health outcomes, and Table 3 shows the Pearson’s correlation for other study variables (continuous variables). Supplementary Table S1 presents the comparison of means for continuous outcomes (cumulative responses to crisis variables) and mental health outcomes between groups distinguished based on gender, sexual orientation, and financial situation. Out of the 403 young adults we surveyed, 6% indicated that they were directly (personally) influenced by all four types of crises under study, which included the COVID-19 pandemic, the conflict in Ukraine, the economic downturn, and climate emergency (being personally influenced constitutes the most intensive response in terms of the sense of proximity to the crises). Roughly a quarter (25.3%) reported they were directly affected by three out of the four crises, while 32.3% were affected by two, and 24.6% by just one crisis. There were 11.9% who did not report being directly affected by any crisis. When it came to crisis-induced stress, only 2.0% of the participants reported experiencing high stress levels due to all the examined crises. Meanwhile, 4.2% indicated high stress levels caused by three crises, 18.6% by two crises, and 31.0% by one crisis. Notably, a significant percentage (44.2%) did not report experiencing high stress levels due to any of the crises. As for the sense of responsibility toward the crises, most respondents did not report feeling a high level of responsibility for any of the crises. Nonetheless, 24.6% of respondents felt strongly responsible for mitigating one crisis, 11.2% for two crises, 1.5% for three crises, and a small fraction (1.3%) felt a high level of responsibility for mitigating all the crises. In terms of experiencing everyday moral dilemmas, 1.0% of the surveyed college students reported such daily experiences in relation to all four crises. Meanwhile, 0.5% related them to three crises, 4.0% to two crises, 17.4% to one crisis, and a substantial 77.2% reported no everyday moral dilemmas concerning any of the crises.

**Table 1:** Descriptive statistics for the individual psychological responses to the crises (N = 403).

Crisis	Response option			
Sense of proximity	I have never heard	Known only from the media	Someone I know was affected	I have been affected/experienced it
Proximity of the Covid-19 pandemic	0.74	4.96	26.30	67.99
Proximity of the war in Ukraine	0.50	34.24	53.10	12.16
Proximity of the economic crisis	0.50	16.38	18.86	64.27
Proximity of the ecological crisis	1.24	47.39	6.95	44.42
Stress	Not at all stressful	Rather not stressful	Stressful	Very stressful
Stress due to the Covid-19 pandemic	15.63	47.15	29.28	7.94
Stress due to the war in Ukraine	4.71	23.08	49.63	22.58
Stress due to the economic crisis	2.48	13.65	48.64	35.24
Stress due to the ecological crisis	6.95	23.08	46.9	23.08
Sense of responsibility	I do not feel responsible at all	I rather do not feel responsible	I feel somewhat responsible	I feel highly responsible
Responsibility for the Covid-19 pandemic	15.63	35.73	40.2	8.44
Responsibility for the war in Ukraine	10.67	34.24	43.42	11.66
Responsibility for the economic crisis	14.39	42.68	34.74	8.19
Responsibility for to the ecological crisis	7.2	19.6	45.16	28.04
Experiencing everyday moral dilemmas	Never	Rarely	Often	Daily
Everyday moral dilemmas related to the Covid-19 pandemic	32.01	49.88	16.63	1.49
Everyday moral dilemmas related to the war in Ukraine	27.3	44.17	24.07	4.47
Everyday moral dilemmas related to the economic crisis	24.81	33.25	30.77	11.17
Everyday moral dilemmas related to the ecological crisis	14.64	31.27	40.45	13.65

**Table 2:** Descriptive statistics for cumulative psychological responses to the crises and mental health variables.

Variable	Range	Mean	SD	p25	Median	p75	Skewness
Sense of proximity to crises	4–16	12.80	1.99	11.00	13.00	14.00	− 0.44
Stress due to crises	4–16	11.22	2.21	10.00	12.00	13.00	− 0.51
Sense of responsibility for crises	4–16	10.28	2.38	9.00	11.00	12.00	− 0.38
Everyday moral dilemmas related to crises	4–16	8.75	2.56	7.00	9.00	10.00	0.02
Negative affect	1–5	3.35	0.78	2.92	3.33	3.92	− 0.38
Depression symptoms	0–4	3.48	1.38	2.50	3.50	4.33	0.14
Self-assessment of physical & mental health	0–10	5.94	1.80	5.00	6.00	7.50	− 0.33

**Table 3:** Pearson’s correlations between the variables (N = 403).

		1	2	3	4	5	6	7
1	Sense of proximity to crises	1.00						
2	Stress due to crises	0.44***	1.00					
3	Sense of responsibility for mitigating crises	0.36***	0.54***	1.00				
4	Everyday moral dilemmas related to crises	0.31***	0.56***	0.61***	1.00			
5	Negative affect	0.15***	0.27***	0.08	0.15***	1.00		
6	Depression symptoms	0.17***	0.19***	0.11*	0.20***	0.55***	1.00	
7	Self-assessment of physical & mental health	− 0.21***	− 0.25***	− 0.09	− 0.17***	− 0.52***	− 0.54***	1.00
8	Financial situation	− 0.16**	− 0.27***	− 0.15**	− 0.16**	− 0.11*	− 0.13*	0.21***

*p < 0.05; **p < 0.01; ***p < 0.001.

The incidence of concurrent psychological responses related to multiple crises significantly increased when more than just the most intense response was considered. When the two most intense response options were taken into account, 36.2% of the surveyed students reported a sense of proximity for all four crises. Furthermore, 24.3% of students reported feeling stressed or extremely stressed by all four crises. The feeling of being somewhat responsible or very much responsible for mitigating the crises was expressed by 19.3% of the students. Additionally, 7.2% of the respondents reported experiencing everyday moral dilemmas related to all four crises at least often. Detailed statistics are presented in Supplementary Table S2 in the Supplementary Material.

The results from the logit regression model for 16 individual crisis-related outcomes are presented in Table S3 in the Supplementary Material. Odds ratios (OR) along with 95% confidence intervals (CI) and *p*-values were provided for each predictor variable. Females had significantly higher odds of experiencing (a) COVID-related stress (OR = 2.156, *p* = 0.007), (b) war-related stress (OR = 2.003, *p* = 0.015), and (c) climate change-related stress, compared to males. They also had significantly higher odds of feeling responsible for mitigating the climate crisis (OR = 2.204, *p* = 0.005), and of experiencing everyday moral dilemmas related to the climate crisis (OR = 2.240, *p* = 0.005) and war (OR = 1.832, *p* = 0.038).

Other gender individuals had significantly lower odds (OR = 0.223, *p* = 0.023) of the sense of proximity of the COVID-19 crisis compared to males. They also had significantly higher odds of experiencing economic crisis-related stress (OR = 5.658, *p* = 0.014) and everyday moral dilemmas related to the economic crisis (OR = 6.787, *p* = 0.004), compared to males and females.

Non-heterosexual individuals had significantly higher odds of the sense of proximity of the (a) COVID-19 crisis (OR = 2.119, *p* = 0.010), (b) economic crisis (OR = 2.016, *p* = 0.009), (c) climate crisis (OR = 1.938, *p* = 0.006), as well as higher odds of stress related to the climate crisis (OR = 1.605, *p* = 0.037), compared to heterosexual individuals. They also had significantly higher odds of feeling responsible for mitigating climate change (OR = 2.859, *p* = 0.000) and experiencing everyday moral dilemmas related to the climate crisis (OR = 2.333, *p* = 0.000).

Individuals in an unfavorable financial situation had significantly higher odds of (a) the sense of proximity of war (OR = 1.707, *p* = 0.000) and economic crisis (OR = 2.701, *p* = 0.000); (b) stress related to COVID-19 (OR = 2.543, *p* = 0.000), war (OR = 1.695, *p* = 0.000), and the economic crisis (OR = 3.226, *p* = 0.000); (c) feeling responsible for mitigating the COVID-19 crisis (OR = 1.701, *p* = 0.015); and (d) experiencing everyday moral dilemmas related to the COVID-19 crisis (OR = 1.840, *p* = 0.007) and economic crisis (OR = 1.836, *p* = 0.005).

The logistic regression analysis indicated that there were several significant associations between self-reported physical and mental health status and (a) the sense of proximity of the economic crisis (OR = 0.731, *p* = 0.000), (b) stress related to COVID-19 (OR = 0.869, *p* = 0.050) and the economic crisis (OR = 0.730, *p* = 0.000), and (c) sense of responsibility for mitigating the economic crisis (OR = 0.841, *p* = 0.016), after controlling for other relevant variables. Better self-reported health was associated with lower odds of reporting the above-mentioned outcomes. Negative affect was significantly associated with stress related to war (OR = 1.725, *p* = 0.001). Depression was significantly associated with the sense of proximity of COVID-19 (OR = 1. 275, *p* = 0.025) and climate change-related stress (OR = 1. 348, *p* = 0.001).

Analysis using a linear regression model revealed significant associations between gender, sexual orientation, financial situation, health status, and different dimensions of four cumulative crisis outcomes (Table 4). Being female was positively associated with heightened stress levels, a sense of responsibility, and experiencing everyday moral dilemmas related to the cumulative crises. Individuals with a non-heterosexual orientation reported an increased sense of proximity and responsibility for mitigating cumulative crises. Experiencing an unfavorable financial situation was also significantly associated with higher stress levels, a greater sense of proximity, and responsibility for mitigating cumulative crises. Individuals with better self-reported health tended to report lower stress and perceived proximity of cumulative crises. Depression showed a positive association with the sense of responsibility for mitigating cumulative crises. Negative affect was not associated with any type of response to the cumulative crises. The analysis revealed that gender, sexual orientation, financial situation, and self-assessed health status collectively accounted for a significant proportion of the variance in the psychological responses to crisis perceptions and improved the models’ predictive accuracy. As indicated by the R-squared values ranging from 0.059 to 0.174 (Table 4), the included predictors explained between 5.9 and 17.4% of the variability in cumulative crisis outcomes across different dimensions.

**Table 4:** Associations between psychological responses for crises and demographic and mental health variables (N = 371).

Descriptive variables	Sense of proximity to crises	Stress due to crises	Sense of responsibility for crises	Everyday moral dilemmas related to crises
	β	β	β	β
	95% CI	95% CI	95% CI	95% CI
	p-value	p-value	p-value	p-value
Gender (ref. = male)				
Female	1.204	3.380***	2.620**	2.678**
	(0.670–2.164)	(1.827–6.254)	(1.299–5.283)	(1.269–5.651)
	0.533	< .001	0.007	0.010
Other	0.588	2.086	1.248	2.520
	(0.173–1.993)	(0.579–7.516)	(0.289–5.379)	(0.532–11.94)
	0.393	0.260	0.766	0.244
Sexual orientation (ref. = heterosexual)				
Non-heterosexual	2.161**	1.153	1.923*	1.440
	(1.368–3.412)	(0.713–1.862)	(1.113–3.323)	(0.805–2.578)
	0.001	0.561	0.019	0.219
Financial situation (ref. = favorable)				
Unfavorable	1.633*	3.028***	1.691	1.864*
	(1.048–2.546)	(1.900–4.826)	(0.994–2.878)	(1.059–3.282)
	0.031	< .001	0.053	0.031
Self-assessment of physical and mental health	0.845*	0.809**	0.903	0.882
	(0.731–0.976)	(0.695–0.942)	(0.760–1.074)	(0.734–1.061)
	0.022	0.006	0.249	0.182
Negative affect	0.977	1.299	0.862	0.908
	(0.702–1.360)	(0.918–1.838)	(0.580–1.281)	(0.595–1.383)
	0.892	0.140	0.461	0.651
Depression symptoms	1.089	1.001	1.091	1.289*
	(0.905–1.311)	(0.824–1.216)	(0.874–1.363)	(1.018–1.633)
	0.367	0.992	0.439	0.0352
Constant	488,583***	27,037***	21,667***	2449***
	(91,326–2,614,000)	(4650–157,204)	(2913–161,186)	(289.1–20,739)
	0	0	0	0
R-squared	0.093	0.174	0.059	0.081

## Discussion

This study aimed to determine the extent to which four co-occurring crises (COVID-19, war in Ukraine, economic crisis, and ecological crisis) affected young adults (being college students) in Poland. We were particularly interested in four distinct areas of the psychological response to polycrisis: sense of proximity, stress caused, sense of responsibility for mitigating the crises, and everyday moral dilemmas related to them (while sense of responsibility and experiencing everyday moral dilemmas may account for a comprehensive area of moral stress).

We supplemented this evidence by showing a co-occurrence of psychological responses to COVID-19, war in Ukraine, economic crisis, and ecological crises in all four areas of response to the polycrisis. This suggests that if young adults (from our sample of college students) exhibit a psychological response to any crisis, it is more likely to be a reaction to multiple crises simultaneously. For instance, when individuals report stress related to a crisis, it is more common for them to experience stress stemming from two, three, or even four concurrent crises. It may be interpreted as initial evidence for the polycrisis being associated with cumulative stress constituted by interrelated stressors, and not only a potential source of several distinct stressors. In the scholarship on crises, it is argued that various concurrent global and local crises interact and reinforce each other; their emergent character constitutes a polycrisis and calls for special measures^4^. Consequently, the psychological response to this situation can be constituted by interacting and mutually reinforcing stressors, in contrast to single stressors induced by individual crises. This calls for further research, which can nevertheless draw inspiration from existing scholarship on cumulative stress both related and unrelated to crises, e.g. Refs.^30–34^. This not only suggests that various environmental (and personal) stressors accumulate and contribute to a heightened stress response and negative consequences for the health and well-being of an individual, but also that the accumulation of stressors may mitigate the protective effects of certain personal characteristics and other resources that enable positive stress coping^30^. Additionally, in young people (including young adults), the accumulation of stressors may prevent the ongoing development of positive stress coping mechanisms^30^^,^^68^. Taking into account the universality of environmental stressors associated with local and global crises, the cumulative stress associated with polycrisis seems to be of relevance to various stakeholders, including public health scholars and policymakers involved in the issue of population resilience in times of the polycrisis. When discussing public health perspectives, it is important to consider the indirect costs associated with the mental health consequences of cumulative stress among young people growing up during times of polycrisis.

We focused on college students from disadvantaged groups (i.e. females and other gender individuals, those with a non-heterosexual orientation, and those with an unfavorable economic situation). We showed that these individuals reported a stronger negative psychological response in all examined areas compared with other college students. Our results—presented in a new context of cumulative stress related to polycrisis—corroborate prior findings that emphasize that individuals from vulnerable groups may be more prone to stress and mental health deterioration in the face of various singular^29^^,^^69–74^ and interrelated crises, including the climate crisis and cost-of-living crisis^12^^,^^16^^,^^54^^,^^75^^,^^76^.

Our results also showed that young people from disadvantaged groups experienced higher levels of negative affect and depressive symptoms and worse self-evaluated physical and mental health. As expected, in our study, the difficult financial situation of young adults comprised a vulnerability factor for mental health deterioration in all stress areas and dimensions of mental health examined. This finding was in line with prior studies showing that lower economic status was the most unquestionable and universal predictor of stress and deteriorated mental health^29^^,^^77–80^. We confirmed its significance in relation to the cumulative stress of the polycrisis experienced by young adults (being college students). This, in turn, implies that economic status, along with other vulnerability factors, should be addressed when designing tailored mental health interventions related to a polycrisis in Poland and Europe^17^. Next, our results showing that male college students in Poland experience more favorable mental health conditions than females and other genders, corroborate previous findings on the associations between gender and stress. They showed that women, including young women, have higher levels of stress and decreased mental health due to a combination of biological and social determinants, more stressful life events, more responsibilities and social demands toward their various life roles, and a greater risk of marginalization^39^^,^^81^. Furthermore, women and girls experience harassment, discrimination, and violence more frequently than men^81–84^. They also have a systematically lower socioeconomic status and limited access to various resources^85–88^. This is why the female gender is one of the criteria for social disadvantage, which may also be a reason for women experiencing minority stress, especially when belonging to other minority groups^89^.

Minority stress (alongside the burden and trauma resulting from experiences of discrimination and violence) helps explain mental health disparities, especially among gender and sexual orientation minority groups^15^^,^^38^^,^^41–45^^,^^48–52^^,^^90^. Although we did not directly measure minority stress in our study, we hypothesize that minority stress may add to the cumulative stress and partly explain worse mental health conditions and higher stress levels in young adults (being college students), namely females and other genders and individuals with non-heterosexual orientations, as shown in our study. However, this hypothesis should be verified in future studies. Future studies should also explore the mechanisms underlying this assumed cumulative effect of minority stress and psychological responses to polycrisis. This would help understand the complexity regarding the vulnerability of disadvantaged young people to climate change-related mental health impacts^12^. The most likely explanations may be based on increasingly scarce resources to which disadvantaged groups have even more difficult access, and on psychological evidence that various stressors in life are cumulative^68^^,^^91–93^. Importantly, the psychological understanding of various life and environmental stressors includes an individual’s subjective evaluation of events and biological readiness to react in a distressed way^94^.

However, the sensitizing aspect of awareness and a value-based sense of responsibility may also be considered in this context^12^^,^^23^^,^^24^. Previous research on lesbian, gay, bisexual, and transgender (LGBT) and feminist activism explains the awareness and sensitivity of non-heteronormative individuals, and their involvement in working for the LGBT community and supporting a more general cause of equity and social justice^95^^,^^96^. Although such activism is only a specific expression of a sense of responsibility for social causes, our results indicated that a general sense of responsibility for mitigating crises and moral dilemmas in the face of everyday decision-making related to crises was stronger and more pronounced in disadvantaged groups of the examined young people (being college students), which was in line with the aforementioned findings. Additionally, minority stress, like any other type of stress, may psychologically sensitize young people to become more aware of and concerned about local and global crisis situations that induce a threat of suffering and injustice. Consequently, their stress levels, including moral stress, may be exacerbated. These issues should be verified in future research and subsequently be discussed in the context of mental health interventions and prevention, which may be of relevance for various stakeholders, including mental health practitioners (psychologists, psychotherapists, medical doctors etc.), social workers, and educators. Importantly, the misinterpretation of gender and sexual orientation as simple predictors of mental health must be avoided. Being a female and of other gender, and having a non-heterosexual orientation were associated with a greater burden of stress and suffering, and may contribute to worse mental health (based on the mechanisms addressed above); linking them unequivocally to mental symptoms and disorders would be false and perpetuate discrimination. Further application of the results should reflect how to support young people from disadvantaged groups in their awareness, sensitivity, and involvement in working for various social and environmental causes, to protect their mental health and ensure that mental health and educational interventions are just.

## Conclusions

This research aimed to examine whether the impact of polycrisis on mental health is greater in young adults from disadvantaged groups (females and other genders, individuals with non-heterosexual orientation, and individuals in unfavorable financial situation) than in other young adults. Our results revealed a greater impact on disadvantaged young adults in four areas: sense of proximity to the crises, stress caused by them, sense of responsibility for mitigating them, and everyday moral dilemmas regarding them. They also suffered more in terms of negative affectivity, depressive symptoms, and subjective physical and mental health.

### Limitations of the study

This study had certain limitations that are discussed in this section. However, the study is part of a larger project that aims to examine psychosocial and clinical aspects of the cumulative stress of polycrisis among young people in Poland. The results presented in this paper come from the pilot stage of the larger project and explore the issue, while providing first evidence that serves to determine directions for further research. Therefore, future data will be collected and additional evidence will be presented, and the current limitations of the study shall be addressed.

First, despite being common in pilot studies, the use of a convenience sample is a limitation of the study compared to using a representative sample of college students or young adults in Poland. The use of cross-sectional data prevented the formulation of causal conclusions. In particular, the results of higher levels of negative affect, depression symptoms, and worse self-evaluated physical and mental health in college students from disadvantaged groups are difficult to unambiguously interpret as being linked to polycrisis rather than the general unfavorable situation of these groups. Additional data collection tools and more advanced analyses are required to obtain conclusive results in this respect; for example, the mediating role of generalized anxiety and depressive symptoms in experiencing the cumulative stress of polycrisis should be addressed. Furthermore, subsequent studies can focus on distinct areas of cumulative stress (i.e. sense of proximity to crises, crises-related stress, and moral stress related to crises) and their specific associations with mental health. Collecting longitudinal data may enable the identification of the causes and consequences of cumulative stress of polycrisis. Moreover, including qualitative analysis in subsequent studies would help go beyond self-reported data and allow for a better understanding of the specificity of the psychological response to polycrisis in young adults. As a sense of responsibility and everyday experiences of moral dilemmas were addressed as elements of crises-related moral stress in young adults, it would be interesting to approach them from a theoretical perspective of moral developmental psychology.

Second, this study examined a limited number of mental health measures. Including other measures of mental health (e.g. symptoms of anxiety^65^), well-being, and human flourishing^59^ would be beneficial. Not only would it provide evidence on mental health risk factors, but also on potential protective factors in disadvantaged young adults.

Third, this study examined only selected groups of disadvantaged young adults. Future studies should address the psychological response to polycrisis in individuals with pre-existing mental health conditions, which might determine the psychological response to the burden of polycrisis in various ways^97^^,^^98^. Another group worth considering is individuals with migrant and refugee backgrounds. In Poland, a comparatively monoethnic country, the war and earlier unrest in Ukraine have resulted in considerable migrant and refugee inflows (including many young people) since 2014^99^^,^^100^. Additionally, young people from rural areas compared with those of urban residence and origin are worth examining^101^^,^^102^. Finally, this study examined only a selected group of college students. Although they were from various universities, faculties, and cities in Poland, our results can be interpreted only concerning the young people who participated in our study. Furthermore, higher education has been recognized as a protective factor against poor mental health. While the level of higher education in Poland is high, with most individuals aged 18–24 studying at universities or enrolled in higher education institutions^103^, looking beyond this group in a future investigation of young adults’ mental health, stress, and well-being will certainly provide more generalized results.

### Supplementary Information

Supplementary Tables.

## Data Availability

Data used for this study are stored in Jagiellonian University Repository and may be available upon request. If someone wants to request the data from this study, please contact Bernadetta Izydorczyk.
