# Supplementary material for: Young adults from disadvantaged groups experience more stress and deterioration in mental health associated with polycrisis
Source: Sci Rep. 2024 Apr 16;14:8757. doi: 10.1038/s41598-024-59325-8 (PMC11021532; doi:10.1038/s41598-024-59325-8)
Supplement: Supplementary file 1 — Supplementary Tables. [file 41598_2024_59325_MOESM1_ESM.docx]

**SUPPLEMENTARY MATERIALS**

**Title: Young adults from disadvantaged groups experience more stress and deterioration in mental health associated with polycrisis**

Weronika Kałwak^1,*^, Dorota Weziak-Bialowolska^2,3^, Anna Wendołowska^1^, Karolina Bonarska^1^, Katarzyna Sitnik-Warchulska^4^, Anna Bańbura^1^, Dorota Czyżowska^1^, Aleksandra Gruszka^1^, Małgorzata Opoczyńska-Morasiewicz^1^, Bernadetta Izydorczyk^1^

^1^ Institute of Psychology, Faculty of Philosophy, Jagiellonian University, ul. Romana Ingardena 6, 30-060 Kraków, Poland

^2^ Department of Quantitative Methods and Information Technology, Kozminski University, ul. Jagiellonska 57/59, 03-301Warsaw, Poland

^3^ Human Flourishing Program, Institute for Quantitative Social Science, Harvard University, 12 Arrow St, Cambridge, 02138 MA, USA

^4^ Institute of Applied Psychology, Faculty of Management and Social Communication, Jagiellonian University, ul. Prof. Stefana Łojasiewicza 4, 30-348 Kraków, Poland

* Corresponding author:

Weronika Kałwak
Institute of Psychology, Faculty of Philosophy, Jagiellonian University, ul. Romana Ingardena 6, 30-060 Kraków, Poland

weronika.kalwak@uj.edu.pl

Table S1. Descriptive statistics by gender, sexual orientation, and financial status (N=403)

|  | Gender | | | | |  | | | Sexual orientation | | | | |  | | | Financial situation | | | | |  |  |  |
| --- | --- | --- | --- | --- | --- | --- | --- | --- | --- | --- | --- | --- | --- | --- | --- | --- | --- | --- | --- | --- | --- | --- | --- | --- |
|  | Male  (*n*=64)  *M/SD* | | | Other  (*n*=339)  *M/SD* | | | Gender differences  Two-sided t-test | | | Non-heterosexual (*n*=119)  *M/SD* | | Heterosexual  (*n*=284)  M/SD | | | Sexual orientation differences  Two-sided t-test | | | Difficult  (*n*=121)  *M/SD* | | Favorable  (*n*=282)  *M/SD* | | Financial situation differences  Two-sided t-test | |  |
| Sense of proximity to crises (4–16) | 12.56/ 2.06 |  | 12.84, 1.98 | | -1.01 | | | 13.38/ 1.84 | | | 12.56/ 2.01 | | 3.98*** | | | 13.21/1.96 | | | 12.62/1.99 | | -2.78** | | | |
| Stress due to crises (4–16) | 10.17/ 1.95 |  | 11.42/2.20 | | -4.61*** | | | 11.39/ 1.99 | | | 11.15/ 2.29 | | 1.02 | | | 12.15/1.99 | | | 10.83/2.18 | | -5.94*** | | | |
| Sense of responsibility for crises (4–16) | 9.47/ 2.03 |  | 10.44/ 2.42 | | -3.39** | | | 10.71/2.30 | | | 10.10/ 2.40 | | 2.41* | | | 10.75/2.34 | | | 10.08/2.38 | | -2.62** | | | |
| Everyday moral dilemmas related to crises (4–16) | 8.00/ 2.34 |  | 8.89/ 2.58 | | -2.73** | | | 9.10/ 8.61 | | | 8.60/ 8.30 | | 1.75 | | | 9.35/ 8.87 | | | 8.48/ 2.45 | | -3.03** | | | |
| Moral stress related to crises (8–32) | 17.47/ 3.75 |  | 19.32/ 4.50 | | -3.51*** | | | 19.82/ 4.25 | | | 18.70/4.48 | | 2.36* | | | 20.11/ 4.59 | | | 18.57/ 4.29 | | -3.15** | | | |
| Negative affect (1–5) | 3.00/ 0.84 |  | 3.42/ 0.76 | | -3.71*** | | | 3.49/.77 | | | 3.30/.78 | | 2.32* | | | 3.53/.77 | | | 3.28/.78 | | -2.92** | | | |
| Depression symptoms (0–4) | 3.21/ 1.39 |  | 3.53/ 1.38 | | -1.59 | | | 3.84/1.43 | | | 3.33/1.33 | | 3.19** | | | 3.77/1.50 | | | 3.35/1.31 | | -2.54* | | | |
| Self-assessment of physical & mental health (0–10) | 5.88/ 1.82 |  | 5.95/ 1.80 | | -0.29 | | | 5.46/1.84 | | | 6.15/1.80 | | -3.48*** | | | 5.48/1.97 | | | 6.14/1.63 | | 3.27** | | | |

M, mean; SD, standard deviation; **p*<.05; ***p*<.01; ****p*<.001.

Table S2. Incidence of concurrent psychological responses (in %) related to COVID-19, war in Ukraine, economic, and ecological crises (N=403)

| Area | Number of crises (%) | | | | |  |
| --- | --- | --- | --- | --- | --- | --- |
|  | 0 | 1 | 2 | 3 | 4 | |
| Sense of proximity (only the most affected/extreme response) | 11.9 | 24.6 | 32.3 | 25.3 | 6.0 | |
| Stress (only the most affected/extreme response) | 44.2 | 31.0 | 18.6 | 4.2 | 2.0 | |
| Sense of responsibility (only the most affected/extreme response) | 61.5 | 24.6 | 11.2 | 1.5 | 1.2 | |
| Everyday moral dilemmas (only the most affected/extreme response) | 77.2 | 17.4 | 4.0 | 0.5 | 1.0 | |
| Sense of proximity (two last extreme responses) | 1.7 | 8.4 | 20.1 | 33.5 | 36.2 | |
| Stress (two last extreme responses) | 5.7 | 11.7 | 20.6 | 37.7 | 24.3 | |
| Sense of responsibility (two last extreme responses) | 12.7 | 16.9 | 27.8 | 23.3 | 19.4 | |
| Everyday moral dilemmas (two last extreme responses) | 28.0 | 26.8 | 26.8 | 11.2 | 7.2 | |

Table S3. Logit regression models for individual crisis-related outcomes

|  | Sense of proximity to | | | | Stress related to | | | | Sense of responsibility for | | | | Everyday moral dilemmas related to | | | |
| --- | --- | --- | --- | --- | --- | --- | --- | --- | --- | --- | --- | --- | --- | --- | --- | --- |
|  | COVID-19 | war | economic crisis | climate crisis | COVID-19 | war | economic crisis | climate crisis | COVID-19 | war | economic crisis | climate crisis | COVID-19 | war | economic crisis | climate crisis |
| Females | 0.965 | 1.145 | 0.879 | 1.604 | 2.156** | 2.003* | 1.489 | 3.071*** | 1.473 | 1.553 | 1.631 | 2.204** | 1.285 | 1.832* | 1.762 | 2.240** |
|  | (0.499-1.865) | (0.640-2.046) | (0.465-1.664) | (0.877-2.934) | (1.230-3.781) | (1.142-3.512) | (0.839-2.644) | (1.744-5.408) | (0.855-2.536) | (0.892-2.704) | (0.913-2.913) | (1.267-3.834) | (0.724-2.282) | (1.034-3.246) | (0.993-3.125) | (1.271-3.950) |
| Other genders | 0.223* | 0.297 | 2.199 | 1.600 | 1.251 | 0.927 | 5.658* | 2.377 | 2.413 | 0.703 | 1.412 | 1.439 | 1.215 | 0.688 | 6.787** | 1.708 |
|  | (0.0610-0.813) | (0.0839-1.049) | (0.406-11.90) | (0.436-5.871) | (0.369-4.241) | (0.264-3.254) | (1.421-22.53) | (0.694-8.141) | (0.657-8.861) | (0.206-2.396) | (0.430-4.637) | (0.379-5.461) | (0.373-3.953) | (0.204-2.323) | (1.847-24.94) | (0.426-6.854) |
| Non-heterosexuals | 2.119** | 1.405 | 2.016** | 1.938** | 0.785 | 1.024 | 1.150 | 1.605* | 0.948 | 1.488 | 0.955 | 2.859*** | 0.842 | 1.073 | 0.941 | 2.333*** |
|  | (1.200-3.742) | (0.892-2.214) | (1.189-3.419) | (1.211-3.101) | (0.508-1.215) | (0.659-1.592) | (0.730-1.811) | (1.029-2.505) | (0.616-1.460) | (0.961-2.305) | (0.618-1.474) | (1.829-4.471) | (0.539-1.316) | (0.690-1.669) | (0.609-1.455) | (1.493-3.644) |
| Economic situation | 0.661 | 1.707* | 2.701*** | 1.348 | 2.543*** | 1.695* | 3.226*** | 1.200 | 1.701* | 1.177 | 1.397 | 1.039 | 1.840** | 1.135 | 1.836** | 0.935 |
|  | (0.405-1.080) | (1.087-2.679) | (1.592-4.581) | (0.863-2.105) | (1.639-3.947) | (1.091-2.634) | (2.033-5.120) | (0.787-1.830) | (1.108-2.613) | (0.769-1.802) | (0.919-2.123) | (0.681-1.585) | (1.177-2.878) | (0.742-1.737) | (1.201-2.807) | (0.612-1.427) |
| Self-assessment of physical & mental health | 0.955 | 1.024 | 0.731*** | 0.865 | 0.869* | 0.967 | 0.730*** | 0.929 | 0.959 | 0.980 | 0.841* | 1.029 | 0.940 | 0.907 | 0.923 | 0.932 |
|  | (0.810-1.125) | (0.886-1.183) | (0.618-0.865) | (0.748-1.000) | (0.755-1.000) | (0.838-1.117) | (0.628-0.848) | (0.809-1.067) | (0.836-1.100) | (0.855-1.123) | (0.730-0.968) | (0.894-1.184) | (0.818-1.080) | (0.792-1.038) | (0.805-1.058) | (0.813-1.069) |
| Negative affect | 1.184 | 1.260 | 0.814 | 0.754 | 0.965 | 1.725** | 1.133 | 1.020 | 1.021 | 1.060 | 0.766 | 0.958 | 0.906 | 0.914 | 1.051 | 1.001 |
|  | (0.813-1.724) | (0.900-1.763) | (0.570-1.163) | (0.534-1.064) | (0.699-1.331) | (1.239-2.402) | (0.815-1.573) | (0.741-1.403) | (0.743-1.403) | (0.768-1.462) | (0.553-1.059) | (0.700-1.312) | (0.654-1.255) | (0.666-1.256) | (0.764-1.445) | (0.725-1.381) |
| Depression | 1.275* | 1.035 | 0.933 | 1.140 | 0.947 | 0.901 | 0.854 | 1.348** | 0.954 | 1.014 | 1.050 | 1.184 | 1.134 | 1.150 | 1.018 | 1.284** |
|  | (1.031-1.578) | (0.859-1.247) | (0.765-1.138) | (0.945-1.375) | (0.788-1.138) | (0.752-1.079) | (0.707-1.033) | (1.122-1.618) | (0.795-1.145) | (0.848-1.212) | (0.874-1.262) | (0.989-1.419) | (0.938-1.370) | (0.963-1.374) | (0.854-1.214) | (1.071-1.540) |
| /cut1 | 0.021*** | 0.0192*** | 0.000*** | 0.004*** | 0.120* | 0.314 | 0.005*** | 0.364 | 0.198* | 0.208 | 0.045*** | 0.320 | 0.483 | 0.426 | 0.502 | 0.558 |
|  | (0.00236-0.185) | (0.00221-0.166) | (3.55e-05-0.00412) | (0.000477-0.0285) | (0.0237-0.605) | (0.0570-1.727) | (0.000865-0.0332) | (0.0711-1.858) | (0.0398-0.981) | (0.0407-1.059) | (0.00866-0.238) | (0.0616-1.665) | (0.0968-2.405) | (0.0887-2.050) | (0.100-2.519) | (0.114-2.724) |
| /cut2 | 0.166 | 1.978 | 0.016*** | 0.467 | 1.262 | 2.728 | 0.047*** | 2.427 | 1.163 | 1.551 | 0.376 | 1.643 | 5.060* | 2.968 | 2.151 | 3.186 |
|  | (0.0246-1.121) | (0.366-10.68) | (0.00232-0.112) | (0.0847-2.572) | (0.255-6.251) | (0.514-14.48) | (0.00857-0.263) | (0.480-12.27) | (0.236-5.718) | (0.309-7.797) | (0.0735-1.926) | (0.320-8.424) | (1.008-25.40) | (0.615-14.32) | (0.428-10.81) | (0.652-15.57) |
| /cut3 | 1.353 | 30.15*** | 0.0476** | 0.615 | 9.454** | 28.12*** | 0.570 | 23.06*** | 12.66** | 14.80** | 3.122 | 13.21** | 85.11*** | 29.13*** | 12.85** | 29.33*** |
|  | (0.206-8.877) | (5.365-169.5) | (0.00700-0.324) | (0.112-3.387) | (1.844-48.46) | (5.105-154.9) | (0.106-3.073) | (4.432-120.0) | (2.501-64.13) | (2.863-76.51) | (0.604-16.13) | (2.512-69.43) | (13.75-526.6) | (5.642-150.4) | (2.505-65.94) | (5.818-147.9) |
| *** p<0.001, ** p<0.01, * p<0.05; N=371  Unstandardized regression coefficients b and 95% CI (in parentheses) are reported | | | | | | | | | | | | | | | | |

Table S3a. Associations between psychological responses to crises and demographic and mental health variables (N=371)

|  | Sense of proximity of the COVID-19 pandemic | Sense of proximity of the war in Ukraine | Sense of proximity of the economic crisis | Sense of proximity of the ecological crisis | Stress due to the COVID-19 pandemic | Stress due to the war in Ukraine | Stress due to the economic crisis | Stress due to the ecological crisis |
| --- | --- | --- | --- | --- | --- | --- | --- | --- |
| Descriptive variables | OR | OR | OR | OR | OR | OR | OR | OR |
|  | 95% CI | 95% CI | 95% CI | 95% CI | 95% CI | 95% CI | 95% CI | 95% CI |
|  | p-value | p-value | p-value | p-value | p-value | p-value | p-value | p-value |
| Gender (ref.=male) |  |  |  |  |  |  |  |  |
| Female | 0.965 | 1.145 | 0.879 | 1.604 | 2.156** | 2.003* | 1.489 | 3.071*** |
|  | (0.499-1.865) | (0.640-2.046) | (0.465-1.664) | (0.877-2.934) | (1.230-3.781) | (1.142-3.512) | (0.839-2.644) | (1.744-5.408) |
|  | 0.915 | 0.649 | 0.692 | 0.125 | 0.007 | 0.015 | 0.173 | <.001 |
| Other | 0.223* | 0.297 | 2.199 | 1.600 | 1.251 | 0.927 | 5.658* | 2.377 |
|  | (0.061-0.813) | (0.084-1.049) | (0.406-11.90) | (0.436-5.871) | (0.369-4.241) | (0.264-3.254) | (1.421-22.53) | (0.694-8.141) |
|  | 0.0230 | 0.0593 | 0.360 | 0.478 | 0.719 | 0.906 | 0.014 | 0.168 |
| Sexual orientation (ref. =heteronormative) |  |  |  |  |  |  |  |  |
| Non-heteronormative | 2.119** | 1.405 | 2.016** | 1.938** | 0.785 | 1.024 | 1.150 | 1.605* |
|  | (1.200-3.742) | (0.892-2.214) | (1.189-3.419) | (1.211-3.101) | (0.508-1.215) | (0.659-1.592) | (0.730-1.811) | (1.029-2.505) |
|  | 0.010 | 0.143 | 0.009 | 0.006 | 0.278 | 0.915 | 0.547 | 0.037 |
| Financial situation (ref. =favorable) |  |  |  |  |  |  |  |  |
| Unfavorable | 0.661 | 1.707* | 2.701*** | 1.348 | 2.543*** | 1.695* | 3.226*** | 1.200 |
|  | (0.405-1.080) | (1.087-2.679) | (1.592-4.581) | (0.863-2.105) | (1.639-3.947) | (1.091-2.634) | (2.033-5.120) | (0.787-1.830) |
|  | 0.099 | 0.020 | <.001 | 0.189 | <.001 | 0.019 | <.001 | 0.398 |
| Self-assessment of physical & mental health | 0.955 | 1.024 | 0.731*** | 0.865 | 0.869* | 0.967 | 0.730*** | 0.929 |
|  | (0.810-1.125) | (0.886-1.183) | (0.618-0.865) | (0.748-1.000) | (0.755-1.000) | (0.838-1.117) | (0.628-0.848) | (0.809-1.067) |
|  | 0.581 | 0.747 | <.001 | 0.050 | 0.050 | 0.648 | <.001 | 0.300 |
| Negative affect | 1.184 | 1.260 | 0.814 | 0.754 | 0.965 | 1.725** | 1.133 | 1.020 |
|  | (0.813-1.724) | (0.900-1.763) | (0.570-1.163) | (0.534-1.064) | (0.699-1.331) | (1.239-2.402) | (0.815-1.573) | (0.741-1.403) |
|  | 0.379 | 0.178 | 0.258 | 0.108 | 0.826 | 0.001 | 0.458 | 0.904 |
| Depression | 1.275* | 1.035 | 0.933 | 1.140 | 0.947 | 0.901 | 0.854 | 1.348** |
|  | (1.031-1.578) | (0.859-1.247) | (0.765-1.138) | (0.945-1.375) | (0.788-1.138) | (0.752-1.079) | (0.707-1.033) | (1.122-1.618) |
|  | 0.025 | 0.719 | 0.495 | 0.172 | 0.561 | 0.258 | 0.105 | 0.001 |

Note: OR, odds ratio, *** p<0.001, ** p<0.01, * p<0.05

Table S3b. Associations between psychological responses for crises and demographic and mental health variables (N=371)

|  | Sense of responsibility for the COVID-19 pandemic | Sense of responsibility for the war in Ukraine | Sense of responsibility for the economic crisis | Sense of responsibility for the ecological crisis | Everyday moral dilemmas related to the COVID-19 pandemic | Everyday moral dilemmas related to the war in Ukraine | Everyday moral dilemmas related to the economic crisis | Everyday moral dilemmas related to the ecological crisis |
| --- | --- | --- | --- | --- | --- | --- | --- | --- |
| Descriptive variables | OR | OR | OR | OR | β | β | β | β |
|  | 95% CI | 95% CI | 95% CI | 95% CI | 95% CI | 95% CI | 95% CI | 95% CI |
|  | p-value | p-value | p-value | p-value | p-value | p-value | p-value | p-value |
| Gender (ref.=male) |  |  |  |  |  |  |  |  |
| Female | 1.473 | 1.553 | 1.631 | 2.204** | 1.285 | 1.832* | 1.762 | 2.240** |
|  | (0.855-2.536) | (0.892-2.704) | (0.913-2.913) | (1.267-3.834) | (0.724-2.282) | (1.034-3.246) | (0.993-3.125) | (1.271-3.950) |
|  | 0.163 | 0.119 | 0.098 | 0.005 | 0.392 | 0.038 | 0.053 | 0.005 |
| Other | 2.413 | 0.703 | 1.412 | 1.439 | 1.215 | 0.688 | 6.787** | 1.708 |
|  | (0.657-8.861) | (0.206-2.396) | (0.430-4.637) | (0.379-5.461) | (0.373-3.953) | (0.204-2.323) | (1.847-24.94) | (0.426-6.854) |
|  | 0.184 | 0.573 | 0.570 | 0.592 | 0.746 | 0.547 | 0.004 | 0.450 |
| Sexual orientation (ref. =heteronormative) |  |  |  |  |  |  |  |  |
| Non-heteronormative | 0.948 | 1.488 | 0.955 | 2.859*** | 0.842 | 1.073 | 0.941 | 2.333*** |
|  | (0.616-1.460) | (0.961-2.305) | (0.618-1.474) | (1.829-4.471) | (0.539-1.316) | (0.690-1.669) | (0.609-1.455) | (1.493-3.644) |
|  | 0.809 | 0.075 | 0.834 | <.001 | 0.451 | 0.755 | 0.786 | <.001 |
| Financial situation (ref. =favorable) |  |  |  |  |  |  |  |  |
| Unfavorable | 1.701* | 1.177 | 1.397 | 1.039 | 1.840** | 1.135 | 1.836** | 0.935 |
|  | (1.108-2.613) | (0.769-1.802) | (0.919-2.123) | (0.681-1.585) | (1.177-2.878) | (0.742-1.737) | (1.201-2.807) | (0.612-1.427) |
|  | 0.015 | 0.454 | 0.118 | 0.860 | 0.007 | 0.559 | 0.005 | 0.754 |
| Self-assessment of physical & mental health | 0.959 | 0.980 | 0.841* | 1.029 | 0.940 | 0.907 | 0.923 | 0.932 |
|  | (0.836-1.100) | (0.855-1.123) | (0.730-0.968) | (0.894-1.184) | (0.818-1.080) | (0.792-1.038) | (0.805-1.058) | (0.813-1.069) |
|  | 0.549 | 0.771 | 0.0159 | 0.694 | 0.381 | 0.156 | 0.248 | 0.317 |
| Negative affect | 1.021 | 1.060 | 0.766 | 0.958 | 0.906 | 0.914 | 1.051 | 1.001 |
|  | (0.743-1.403) | (0.768-1.462) | (0.553-1.059) | (0.700-1.312) | (0.654-1.255) | (0.666-1.256) | (0.764-1.445) | (0.725-1.381) |
|  | 0.897 | 0.724 | 0.106 | 0.791 | 0.553 | 0.580 | 0.761 | 0.996 |
| Depression | 0.954 | 1.014 | 1.050 | 1.184 | 1.134 | 1.150 | 1.018 | 1.284** |
|  | (0.795-1.145) | (0.848-1.212) | (0.874-1.262) | (0.989-1.419) | (0.938-1.370) | (0.963-1.374) | (0.854-1.214) | (1.071-1.540) |
|  | 0.615 | 0.881 | 0.603 | 0.066 | 0.194 | 0.123 | 0.844 | 0.007 |
